# An interprofessional approach to shared decision making: an exploratory case study with family caregivers of one IP home care team

**DOI:** 10.1186/1471-2318-14-83

**Published:** 2014-07-02

**Authors:** France Légaré, Dawn Stacey, Nathalie Brière, Hubert Robitaille, Marie-Claude Lord, Sophie Desroches, Renée Drolet

**Affiliations:** 1 Research Centre of the Centre Hospitalier Universitaire de Québec, Hôpital Saint-François d’Assise, 10 de L’Espinay, Room D6-735, Quebec City G1L 3 L5, Canada; 2 Department of Family Medicine and Emergency Medicine, Faculty of Medicine, Université Laval, Quebec City, Canada; 3 School of Nursing, Faculty of Health Sciences, University of Ottawa, Ottawa, Canada; 4 Ottawa Hospital Research Institute, Ottawa, Canada; 5 Centre de santé et de services sociaux de la Vieille-Capitale, Quebec City, Canada; 6 Centre de santé et de services sociaux de Montmagny-L’Islet, Montmagny, Canada; 7 Department of Food Science and Nutrition, Université Laval, Quebec City, Canada

**Keywords:** Shared decision making, Interprofessionalism, Home care, Older adults, Qualitative methods, Case study, Family caregivers

## Abstract

**Background:**

Within the context of an exploratory case study, the authors assessed the perceptions of family caregivers about the decision-making process regarding relocating their relative and about the applicability of an interprofessional approach to shared decision making (IP-SDM). They also assessed perceptions of health professionals and health managers about IP-SDM.

**Methods:**

From November 2010 to October 2011, we worked with one IP home care team dedicated to older adults (the case) from a large primary health care organization in Quebec City, Canada. We identified six of their clients who had faced a decision about whether to stay at home or move to a long-term care facility in the past year and interviewed their family caregivers. We explored the decision-making process they had experienced regarding relocating their relative and their perceptions about the applicability of IP-SDM in this context. Attitudes towards IP-SDM and potential barriers to this approach were explored using a focus group with the participating IP home care team, individual interviews with 8 managers and a survey of 272 health professionals from the primary care organization. A hybrid process of inductive and deductive thematic analysis was used and data were triangulated across all sources.

**Results:**

Family caregivers reported lack of agreement on the nature of the decision to be made, a disconnection between home care services and relatives’ needs, and high cost of long-term care alternatives. Factors influencing their decision included their ability to provide care for their relative. While they felt somewhat supported by the IP home care team, they also felt pressured in the decision. Overall, they did not perceive they had been exposed to IP-SDM but agreed that it was applicable in this context. Results from the survey, focus group and interviews with health professionals and managers indicated they all had a favourable attitude towards IP-SDM but many barriers hampered its implementation in their practice.

**Conclusions:**

The family caregivers in this study did not experience IP-SDM when relocating their relative. Added to results obtained with health professionals and managers, this highlights the need for an effective intervention targeting identified barriers to implementing IP-SDM in this context.

## Background

Home care is the fastest growing sector in health care [1]. As in many developed countries, Canada's population is aging, and its seniors are living longer than ever before. In 2010, adults aged 65 years and older represented about 14% (4.8 million) of all Canadians and this proportion will grow to 25% in 2036 [1,2]. Health professionals must be mobilized to ensure that elderly people and their family caregivers participate actively in decision making about their care and to help them make informed value-based decisions [1-4].

### Older adults facing difficult decisions

One of the hardest decisions that the older adult faces is whether to stay at home or relocate to a long-term care facility [1,2]. Multiple factors influence the decision-making process of clients facing this decision [2,5]. Congruent with the literature on best practices for supporting individuals making difficult health-related decisions [6], key components of effective decision support are communicating balanced and tailored information, clarifying values and preferences [7], and providing emotional support [1], while minimizing sources of undue pressure [8]. And yet, in a recent in-depth assessment of the decision making process among older adults facing a decision regarding a long-term care facility, they felt unsupported, lacked information, and did not feel they had participated fully in the decision [9]. Furthermore, the author called for a change in paradigm: from a “they should be in a care facility” approach (a paternalistic decision making process) to one that emphasizes supporting the older adult in being an active participant in the decision making process (a SDM process) [9].

### Shared decision making: an overview

**Figure 1:**
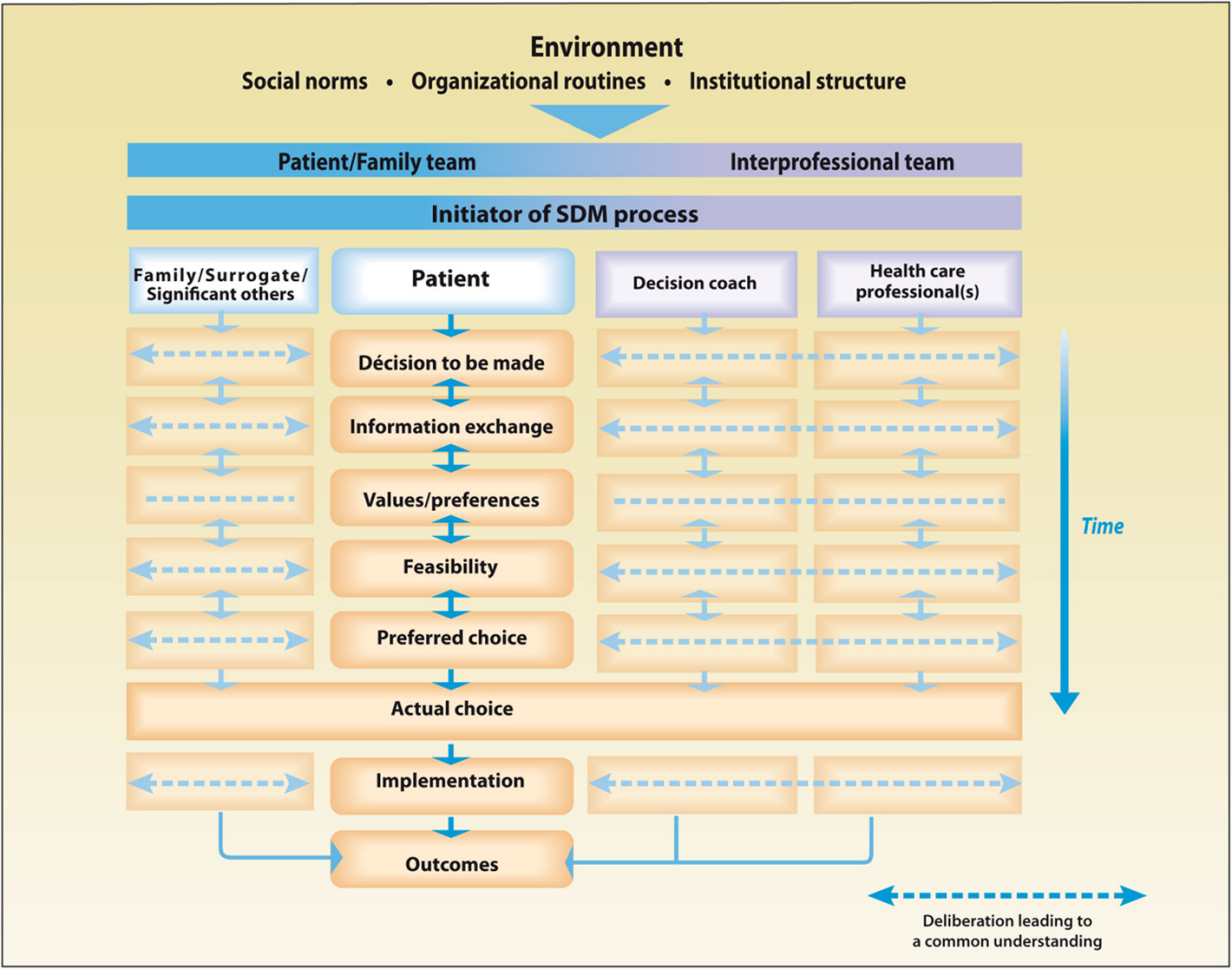
The IP-SDM model.

Shared decision making (SDM) is a process by which health-related decisions are made jointly by the client and his/her health professional and in which both the available evidence and what matters most to the client are used to inform an agreed-upon decision [10]. SDM is typically described as most appropriate for difficult decisions such as those for which clients’ preferences are central to the decision [11-13]. Recently, the concept of SDM has expanded beyond the client/health professional dyad to include significant others such as family members and the interprofessional (IP) healthcare team members. An IP approach to SDM (IP-SDM) provides a structured process for making difficult decisions that takes into account the key components of effective decision support [3,14-16]. Briefly, the model is comprised of two main axes. The vertical axis is the SDM process that occurs over time (identifying the decision to be made, then discussing evidence about the options, clarifying clients’ values, considering the feasibility of each option, and finally reaching consensus on the best option); while the horizontal axis presents the key actors involved in the SDM process, both in the client team (i.e. the client with or without significant others) and in the healthcare team (i.e. two or more healthcare professionals), with the client in the centre of the process (Figure 1) [3,14-16]. The model also includes a decision coach whose role is similar to a care coordinator with a focus on the decision-making process. Elements at the micro level include family members and IP teams; all are situated within broader environmental influences such as the healthcare system and health organizations. The underlying assumption is that involving clients in the SDM process is essential to achieving client-centred care and to reaching decisions that are informed and based on client values and preferences. By achieving a common understanding (horizontal dotted lines) of the SDM steps among all parties involved, and recognizing their various contributions to the process, there will be improved success in reaching a shared decision that is informed by evidence and based on what matters most to clients.

### Research question and objectives of the study

Home care is often delivered by IP teams that may vary in number and may include nurses, occupational therapists, physiotherapists, social workers, dietitians and non-regulated providers [17,18]. However, family caregivers are often the main providers of care for older adults, and although the client is at the centre of the decision about location of care, family caregivers often play a crucial role in the decision [1,19-23] as well as in home care safety generally [24]. Although a few studies have assessed family caregiver perceptions of the decision making process about location of care in diverse healthcare systems [1,19,21,23], we found none that had focused on IP-SDM. This led us to our research question: How is IP-SDM perceived by family caregivers whose relative is under the care of IP home care teams and by health professionals and managers from these home care teams? Consequently, within the context of an exploratory case study aimed at determining the feasibility of implementing IP-SDM in the clinical practice of IP home care teams, we sought to explore the perceptions of family caregivers about the decision-making process they had experienced regarding relocating their relative and about the applicability of IP-SDM in this context. We also assessed opinions of health professionals and managers of IP home care teams about IP-SDM.

## Methods

### Study design

This exploratory case study was embedded in a larger mixed methods study that aimed to assess the feasibility of implementing IP-SDM in the context of home care [15]. This larger mixed methods study included a large quantitative paper-based survey of all the health professionals involved in home care across multiple clinical sectors (e.g. older client care, palliative care, mental care, newborn care, postsurgical care) and for which detailed results have been published [14]. According to the Action cycle of the Knowledge to Action Framework, implementation of new evidence in practice depends on recognizing the gap between current practice and the new knowledge (in the context of this study, IP-SDM), adapting the new knowledge to the local context (in the context of this study, home care), identifying barriers to knowledge use, developing interventions to overcome identified barriers, and monitoring knowledge use, impact, and sustained use [25]. In order to document the opinions of diverse stakeholders including family caregivers on IP-SDM, the research team chose to take a participatory approach that would further mutual respect and collaboration between researchers, the IP home care team and family caregivers. This would in turn increase the ability of the research team to adapt IP-SDM to home care for older adults. In this exploratory case study, the case was the IP home care team dedicated to older adults facing a decision to stay at home or move to a long term care facility, and data was collected from managers, health professionals and family caregivers in the catchment area of the primary care organization.

### Choice of setting

As our overall objective was to determine the feasibility of implementing our IP-SDM model in the clinical practice of IP home care teams, a home care team dedicated to older adults was selected for three reasons. First, the prevalence of chronic age-related diseases is growing, and older adults increasingly require home care [1]. Second, SDM is especially relevant in this setting: older adults and their family caregivers face many complex decisions related to treatment options and may face greater risks linked to healthcare interventions, and thus need to participate more actively in decision-making to make informed value-based decisions. Third, home care teams dedicated to older adults are organized in an IP structure.

### Description of the setting

To preserve anonymity, we have changed the names of the home care unit and locations throughout this paper. Data were collected in the home care program of a large primary care organization covering a population of 290,000 inhabitants of the Québec City area, Canada. In the Province of Québec, these primary care organizations are the result of mergers between local community service centres, long-term care facilities and, in most cases, a hospital. Home care is one of several programs they offer. At the time of the study, the home care programs of this large primary care organization employed 632 part- or full-time employees organized according to specific clienteles such as older client care, palliative care, mental care, newborn care and postsurgical care, with 566 of these employees directly involved in providing home care. The healthcare providers involved were health professionals such as nurses, social workers, occupational therapists, physiotherapists, activity coordinators, dietitians, other social support and rehabilitation workers, and physicians as well as unlicensed home support workers.

### Participants and recruitment procedures

#### Interviews with family caregivers

We performed individual interviews with family caregivers who were receiving services from one IP home care team (the case). We used a convenience sampling strategy to identify participants with help from the IP home care team. Initially we wanted to include older adult clients themselves in order to compare their perspectives with those of their family caregivers on the feasibility of implementing IP-SDM. The Principal Investigator [FL] secured the cooperation of the clinical coordinator of the IP home care team, who contacted potentially eligible participants. Eligibility criteria included being a client (older adult) or a family caregiver for an older adult who: i) was aged 65 years or older; ii) had received care from the IP home care team in the past year; and iii) had faced the decision about whether to stay at home or move to a long-term care facility in the past year. The IP home care team’s workload as well as the severe incapacities of the clients reduced the number of potentially eligible participants to eight family caregivers and clients, who were contacted by a member of the research team [CP]. Six family caregivers agreed to be interviewed. The two clients contacted either did not want to participate or did not have the cognitive capacity. Ethics approval was obtained from the ethics board of the primary care organization, the Centre de Santé et des Services Sociaux de la Vieille Capitale (CSSS-VC) in Quebec City. A consent form was presented and explained to each participant and anonymity and confidentiality were discussed. All participants signed consent forms prior to the interviews. We identified each participant with a code and any names that were mentioned in the interviews were changed to ensure anonymity and confidentiality.

#### Survey, focus group and interviews with managers

In order to obtain a variety of perspectives and to stimulate discussion on IP-SDM, we surveyed all licensed and unlicensed healthcare providers involved in home care in the primary care organization across all clinical sectors (e.g. older client care, palliative care, mental care, newborn care and postsurgical care), and held one focus group with those involved in the IP home care team dedicated to older adults. We also conducted individual interviews with managers at varying levels of influence in the primary care organization including its home care sector.

### Data collection procedures

A senior research assistant trained in healthcare research innovation (CP) conducted individual interviews in French with family caregivers in their homes. She used a semi-structured interview guide created by the research team that was based on key components of IP-SDM [15,26]. She was accompanied by a master’s student in community health who was first trained in health anthropology (GM) and who was asked to provide technical support if needed. The interview sought participants’ experience and perception of IP-SDM. Briefly, each participant was asked about: i) her experience of the decision-making process regarding whether her relative would stay at home or move to a long-term care facility; ii) the role of family caregivers in the decision making process, iii) presentation of the different options by the IP home care team; iv) discussion of clients’ and family caregivers’ values and preferences; v) support or undue pressure from others; and vi) experience and applicability of an interprofessional approach to SDM in this context. To illustrate an ideal case of SDM, a video presenting a clinical scenario based on our IP-SDM model was used to prompt family caregivers answers to the last question (see Table 1). It represented an older woman and her family caregiver (her daughter) engaging with IP home team members in the process of deciding to stay at home or move to a long term care facility. Details on how this video was developed are presented elsewhere [27]. Presenting the video in each interview meant that all participants had a common understanding of IP-SDM, as we were more interested in their opinion about our proposed IP-SDM model than in how they might conceive of IP-SDM. To ensure consistency, all interviews were conducted by the same research assistant (CP) accompanied by the same master’s student (GM) and audio-recorded. The median length of the interviews was 59 minutes (range: 39–96 minutes) including time required to view the 18-minute video. Data collection was stopped when all the questions were covered and participants said they had no other comments to add. All interviews produced a total of 132 pages of transcripts.

**Table 1:** Content of video shown to participants

**Main scene**	**Theme**	**Content**
Introduction (0–2:27)	Presentation of a fictional case: 76 year-old woman with deteriorating health who is living at home with her daughter	Recommendations by an IP home care team about the deterioration of a client’s health
Day 1 Monday (2:28–6:52)	Presentation of options by the social worker to the client assisted by her daughter	The social worker presents two options to the client and her family caregiver based on recommendations by the interdisciplinary team: 1) to adapt the home or 2) to move to a residential facility with services.
		Client has one week to decide.
		Social worker uses a decision aid.
		Social worker does not influence the decision: client has final choice.
Day 2 Tuesday (6:53–10:24)	Team meeting to ensure the follow-up of cases	Social worker relates the situation of the client to her IP home care team comprising a nurse, an occupational therapist and a physiotherapist.
		Each team member evaluates the feasibility of each option in compliance with the client’s choice.
Day 2 Tuesday (10:25–12:10)	Follow up of client case: Nurse calls the client’s family physician	Nurse from the IP home care team relates to the client’s family physician the two options presented to her.
Day 4 Thursday (12:11–14:41)	Physiotherapist’s visit to client’s home	Physiotherapist from the IP home care team asks the client if she has made a decision.
		He evaluates with her the benefits of each option.
		He asks the client if he can transmit the information discussed with her to the social worker.
Day 5 Friday (14:42–15:43)	Follow up of client’s case by the physiotherapist and the social worker	Physiotherapist reports to the social worker the progress of the client’s thinking.
Day 7 Monday (15:44–18:09)	Validation of the selected option	Social worker confirms the decision made by the client in the presence of the family caregiver.

Details of the data collection procedure used with the health professionals, health managers and the IP home care team are described elsewhere [14]. Briefly, we assessed health professionals, and managers’ attitudes, towards IP-SDM as well as their intention to use IP-SDM in their context using a self-administered questionnaire. We also sought the barriers and facilitators they perceived to IP-SDM. Lastly, we conducted one focus group session with the IP-SDM home care team and assessed the barriers and facilitators they perceived to IP-SDM.

### Data analysis

All audio recordings of the family caregivers’ interviews were transcribed by a professional transcriber who was not part of the research team. We used a hybrid process of inductive and deductive thematic analysis. The first categories were identified *a priori* based on the key components of the IP-SDM model. New themes were then identified using a data-driven inductive approach based on deductive *a priori* template of codes approach [28]. Two team members [MCL, MJC] then independently identified themes using an open coding procedure, sorting them into underlying components related to our coding framework [29]. Analysis involved a) reading the full transcripts to obtain a sense of the overall data; b) conducting a thematic analysis using a template of codes based on the key components of an interprofessional approach to SDM and open codes for the new themes that had been inductively derived; and c) comparing coders’ findings to reach agreement about the main themes identified. The analysis was performed using NVivo 9 software (QRS International, Melbourne, Australia). Discrepancies were resolved in discussion with the principal investigator [FL] and HR who read all interview transcripts. Quotes that illustrated the main emergent themes were translated into English by a native English-speaking professional translator. Detailed data analysis methods for the healthcare professionals’ survey, focus group and managers’ interviews are described elsewhere [14]. Triangulation of data to better understand the case was performed by the principal investigators who had diverse backgrounds (FL, a practicing family physician who covers home care for a medical group, DS, a nurse-educator and NB, a health manager in the primary care organization) and the research coordinator (HR).

## Results

The structure of this section is as follows. First, we introduce the findings pertaining to the family caregivers detailing their sociodemographic characteristics and then the main themes identified in the interviews. Second, we briefly report on the findings pertaining to the health professionals’ survey, the IP home care team focus group and the health managers’ interviews. Lastly, we present the key elements of a triangulation of these multiple sources of data.

### Family caregivers’ interviews

Six family caregivers agreed to participate. Table 2 shows their sociodemographic characteristics. All participants were female. Two were caring for a relative diagnosed with Alzheimer’s disease (family caregivers C3 and C6). At the time of the interview, three family caregivers lived with their older relative (in the same house), one family caregiver did not, and two family caregivers had experienced relocation of their relative (from their home to a long-term care facility). Participating family caregivers cared for one older relative each: three women and three men. Family caregivers described many examples of the care that they provide for their relatives (surveillance, bathing, preparing meals, doing housework) and the care they receive from the IP home care team (accompaniment, bathing assistance). These home care services were perceived by family caregivers as reducing the burden of care and as opportunities to leave the house and/or have some free time. In some cases, they mentioned that their relative was also receiving help from private organizations offering services (e.g. accompaniment, daytime supervision). During the interview, participants had the opportunity to narrate their experiences as a family caregiver facing a decision-making process about relocating their relative. Their statements allowed us to identify themes associated with key components of IP-SDM (Table 3). We present these themes and their most significant aspects below.

**Table 2:** Characteristics of participating family caregivers

**Interview**	**Sex**	**Age (years)**	**Kinship or marriage ties with their frail elderly relative**	**Residence ties with their frail elderly relative**	**Sex of their frail elderly relative**
C1	F	74	Spouse	Live in the same house	M
C2	F	60*	Sister	Live in different houses	F
C3	F	61	Daughter	Live in different houses	F
C4	F	55*	Daughter	Live in different houses	F
C5	F	80*	Spouse	Live in the same house	M
C6	F	71	Spouse	Live in the same house	M

*Estimated by the interviewers.

**Table 3:** Perception of family caregivers of the decision-making process about location of care

**Key components of an interprofessional approach to SDM**	**Main themes associated with the key component identified in the interviews**	**Family caregivers**	**Range of quote**	**Quote**
1. Participants’ experience of the decision-making process about location of care	Nature of the decision to be made	**C1**	(1–3)	*“The social worker came when I finally made my relocation request. … she explained everything to me regarding my request for a public facility… she gave me a list of places to visit”. (C3)*
		**C2**		
		**C3**		
		**C4**		
		**C5**		
		**C6**		
	Inability to provide care	**C2**	(3–5)	*“On the week-end it was like I was in jail… without bars but I was in jail”. (C2)*
		**C3**		
				*“What I mean is, I couldn’t really keep her anymore. I had no more patience”. (C3)*
	Inappropriateness of services provided by the home care team	**C1**	(1–4)	*“Home care – yes they come… But it’s not really home care. I’m the one who gives him his shower and takes care of everything”. (C1)*
		**C4**		
		**C5**		
				*“You know, a kind of stick to help him to grab things… that’s not what he needs… If you want to keep old people at home, you have to give them what they want … why is taking a bath less important than putting on support socks? I know that it’s important to wear support socks… but it’s not support socks she needs, she’d like to take a bath”. (C4)*
				*“They don’t listen to us, but we have to listen to them… I think they should listen more to us”. (C5)*
2. Role of client and family caregivers in the decision making process about location of care	Initiating the decision making process	**C1**	(1–4)	*“I’ve always been the one who made the decisions; but I got information about the decision… and then I went looking for a home”. (C1)*
		**C2**		
		**C3**		
		**C4**		
		**C5**		
		**C6**		
				*“When you’re alone in making the decision… I began to search for nursing homes in the phonebook and then I contacted some of them”. (C2)*
	Controlling the information	**C1**	(1–2)	*“We tried to keep it positive. Not say that it’s definite, that he was leaving the house. It’s lying, but it’s lying for a good reason. It’s called a white lie”. (C3)*
		**C4**		
		**C5**		
3. Presentation of the different options by the IP home care team	Not enough options	**C2**	(1–3)	*“It seems there’s a waiting list for getting into a public place… in the meantime she could die, or fall 20 times”. (C2)*
		**C3**		
		**C4**		
		**C5**		
				*“Choices, options—there aren’t that many”. (C3)*
				*“At this time, and given my age, she told me I would be better to ask for a place in a facility right now, because the waiting list is up to two years”. (C5)*
	Too little information	**C2**	(1–4)	*“I had to phone them again and again and we hit a brick wall every time… and then they said that it was not a case for home care services”. (C4)*
		**C4**		
		**C5**		
4. Values and preferences of clients and family caregivers	Differing values among those involved in the decision making process	**C2**	(1–2)	*“She made this decision. It’s hard, as her child, to accept her decisions”. (C2)*
		**C3**		
				*“I completely agree with her decision. After all, it’s her who has to choose”. (C3)*
5. Support or undue pressure from others	Diversity of individuals who were a source of support	**C1**	(1–3)	*“She gave her one week to reflect… It’s not a small decision… it’s her life”. (C1)*
		**C3**		
		**C4**		
	Pressure from the IP home care team	**C1**	(1–3)	*“The more we talk, in any case that’s how it is for me, the more she [the social worker] insists she should stay at home. She keeps saying ‘it’s your choice’ but the fact is, she’s made the decision already”. (C1)*
		**C3**		
		**C4**		
				*“I really didn’t know this system before, but now the government urges us to go private, many more are going to private care. Those who have money can go private… and if you don’t have money, they put you in a public home”. (C3)*
				*“They told me ‘Go to a private home and pay’. But my mother is not a millionaire… And they say that it is not expensive. It costs $1500[Canadian] per month, not everybody can pay that!” (C4)*
6. Experience and applicability of an IP approach to SDM	Lack of experience or exposure to interprofessional work	**C1**	(1–2)	*“There are so many people; it is just like the rehab centre! Because at the rehab centre there are social workers, nurses…” (C1)*
		**C2**		
		**C3**		
		**C4**		
		**C5**		*“She [the social worker] helped me in this way, to focus on her [the client’s] own needs… they’re used to doing this and they used the right words”. (C2)*
		**C6**		
				*“With all the help she’ll get [the physiotherapist, the occupational therapist, the social worker, the nurse and the physician], it seems ideal. If home care was like that everywhere, it would be great”. (C3)*
	Staff turnover as an obstacle to IP approach	**C2**	(1–2)	*“When I called, it was a different social worker”. (C3)*
		**C3**		
		**C4**		*“It’s never the same person, and it’s never at the same time—they change it around when they like”. (C4)*

1. Participants’ experience of the decision-making process about location of care.

All the family caregivers reported that they had discussed the nature of the decision to be made. Interestingly, however, there was more decision support for the process of relocation itself (i.e. to relocate to one long-term care facility compared to another) than for the process leading to the decision to stay home or relocate to a long-term care facility (C1, C2, C3, C4, C5, C6). Reflecting on factors influencing the decision to consider relocating their relative, some family caregivers (C2, C3) mentioned that they no longer felt able to continue providing care for their relative for psychological or physical reasons. This was partly why they decided to consider relocating their relative. In three cases (C1, C4, C5), family caregivers perceived that the home care services provided by the public sector did not respond well to their needs and that the health professionals simply offered them whatever services were available, appropriate or not.

2. Role of client and family caregivers in the decision making process about location of care.

Two main themes emerged: initiating the decision making process and controlling the information. Family caregivers reported having been actively involved in the decision process regarding the location of care of their relatives. Some identified themselves as the initiator of the decision about the localization of care. Two family caregivers (C1, C2) clearly indicated that the clients were not involved in the decision-making process, even though their relatives were not those with Alzheimer’s. Two other family caregivers (C4, C5) explicitly reported some involvement of their relative in the decision-making process but one of the family caregivers (C3) stated that sometimes the truth was not told to their relative.

3. Presentation of the different options by the IP home care team.

Two main themes emerged: not enough options and too little information. Family caregivers (C2, C3, C4, C5) reported that very few options were available to them or that the options were not clearly presented or not properly explained. Availability of beds in long-term care facilities was also reported as a factor to take into account. The same participants mentioned that lack of availability in long-term care facilities in the public sector represents a major obstacle to having any choice about relocating to a care facility.

At the time of making a decision about location of care, family caregivers reported that availability of beds and associated costs were discussed. The cost of relocation to a private facility and the cost of adapting the home of their relative were identified as concerns influencing the decision making process and about which too little information was made available.

4. Values and preferences of clients and family caregivers.

Differing values among those involved in the decision making process was the main theme identified in relation to this component of IP-SDM. Family caregivers were concerned about values and preferences but had difficulty integrating the values of their relative into the decision-making process. Two family caregivers (C2, C3) were not able to continue caring for their relative at home for psychological or physical reasons, in spite of their values and preferences. They also highlighted their difficulty in reconciling the values of their relative with their own needs.

5. Support or undue pressure from others.

Two main themes emerged in relation to this element of IP-SDM: the diversity of individuals who were a source of support, and pressure from the IP home care team. Family caregivers reported receiving support and advice from several individuals including family members (e.g. children, brothers, sisters, cousins) and significant others such as friends and neighbours as well as the social worker in the decision-making process. Some family caregivers (C1, C3, C4) reported that the decision regarding location of care was a very important and sensitive decision and that it should be made after reviewing each option carefully. Some participants (C1, C3, C4) reported pressure from the home care team to relocate their relative to a private facility or else they would have to keep caring for their relative at home.

6. Experiences and applicability of an IP approach to SDM.

In terms of the interprofessional approach portrayed, all family caregivers reported having been supported by only one kind of health professional in the decision making process, namely the social worker. Although participants identified five different health professionals who seemed to be involved in the management of older adults (physicians, nurses, dietitians, social workers and occupational therapists), none had perceived that they worked together. In other words, family caregivers did not have any experience of or exposure to interprofessional work among the many health professionals they had seen involved with their relative, although one family caregiver noted that an interprofessional approach would be helpful. As reported by three family caregivers (C2, C3, C4), staff turnover is also an issue that can compromise the quality of the support, and made follow-up of the file difficult.

### Surveys, focus group and managers’ interviews

Results from the survey of 272 health professionals, one IP home care team focus group and 8 health managers interviews are detailed elsewhere [14]. Briefly, these results indicated they all had a favourable attitude towards IP-SDM and that health professionals intended to engage in IP-SDM in the context of home care. However, many barriers hampered its implementation in their practice. Overall the most frequently mentioned barriers identified by the participants were time constraints, staff workloads, the difficulty of coordinating professionals, failure to synchronize the client care interventions, lack of human resources, high staff turnover, lack of cohesion among professionals in the teams and different work methods and vocabulary. Participants also proposed a few facilitators to help implement IP-SDM in the home care teams, namely, the involvement of all professionals from the outset in the management of a case, provision of tools such as decision aids that are appropriate to an IP-SDM approach, planned team meetings, better team cohesion and sharing of work methods.

### Triangulation of sources of data

Overall, the observations made by family caregivers match the barriers and proposed facilitators to the implementation of IP-SDM reported by health professionals and managers [14]. For example, family caregivers reported that the nature of the decision to be made (decision point) was not always agreed upon by all parties involved, thus validating health professionals’ perception of the difficulty of coordinating diverse professionals in one IP home care team. Also, family caregivers acknowledged that a diversity of individuals supported the decision making process, and perceived pressure by the IP home care team that could be due to the time constraints and high staff turnover earlier identified by the home care staff. Family caregivers also reported having been exposed to high staff turnover. They did not perceive they had been exposed to any interprofessional work, let alone IP-SDM. Nonetheless, they believed that IP-SDM in this context was applicable and indeed desirable, thus validating the health professionals’ support for this idea and their strong intention to engage in IP-SDM in this context.

## Discussion

Within the context of an exploratory case study, the authors assessed the perceptions of family caregivers about the decision-making process regarding relocating their relative and about the applicability of IP-SDM to this context. They also assessed perceptions of health professionals and health managers about IP-SDM. To the best of our knowledge, this is the first study assessing perceptions of family caregivers using a SDM perspective combined with an IP approach. Overall, it indicates that family caregivers, health professionals and health managers shared similar views about IP-SDM and barriers to its implementation in clinical practice. These results lead us to make the following observations.

Firstly, decision support interventions need to help clients, family caregivers and IP home care team members agree initially on the nature of the decision to be made or, in other words, on what decision they are being asked to support. Family caregivers felt that the support provided by the health professional targeted the decision about where to relocate (choosing among care facilities) rather than the decision about relocation or staying home, i.e. they felt supported for a decision subsequent to the initial decision about whether to relocate or stay home. This is important because the nature of the decision, also known as a decision point, sets the stage for all subsequent steps in the decision making process including that of which options should be presented and discussed [30]. This may explain why no family caregivers reported having had all options presented to them (for example, the options for relocation, home care adjustment, home adaptation), or reported having too little information about the options: they had not been informed adequately to make a quality health decision about relocation because a decision point had not been identified [31]. The proposed interprofessional approach to SDM addresses these issues by indicating clearly the need for an agreed upon decision point (horizontal dotted lines) by all parties involved as a first step (decision to be made).

Secondly, it was both reassuring and worrisome to hear family caregivers assuming the role of initiators of the decision making process while at the same time acknowledging that they were in a position of controlling what information to share with the client. The significant role of family caregivers as initiators of the decision making process about location of care has been documented before [1]. However, our study findings provide additional insight. From an SDM perspective, family caregivers’ concerns about sharing information with their relative amount to a paternalistic decision making model. Although health professionals and family caregivers hold strong views regarding which clients want to, should, or even can engage in SDM, those views may be flawed. Surveys consistently indicate that clients want more engagement than they get [32] and this proportion is growing over time [33]. Yet vulnerable populations—such as older people, immigrants, and people with less education in general and those with lower numeracy—report less interest in SDM than other groups [34] and thus are less likely to be invited by their health providers and their family caregivers to be actively engaged in decisions regarding their health. This calls for careful consideration, as vulnerable clients stand to benefit most from engaging in SDM. In fact, the preferred role in decision making represents a set of specific communication behaviors that are modifiable [35]. Indeed, like health professionals, vulnerable clients can learn communication skills and become increasingly confident in their ability to engage in decisions about their health [36] and, in this case, in the location of their care. In other words, given the evidence suggesting that SDM provides optimal care, ethical and moral principles require that IP-SDM should not be withheld from vulnerable clients just because it may be more difficult to deliver it to them. Rather, ways to deliver such care across the board must be found [12].

Thirdly, we encountered family caregivers who reported differing values from those of the IP home care teams involved in the decision making process. They also reported on the fact that there was little consideration of the values and preferences of the client. A common feature in the interviews was that the family caregivers were unanimous about the central role that the client should play in the decision process. This is in complete agreement with SDM and client-centred care that is promoted by our IP-SDM model. Yet family caregivers were deeply ambivalent about reconciling their own values and preferences with those of their relatives and we can hypothesize with those of the IP home care team. It is worrisome that family caregivers were concerned about the values and preferences of their relative but admitted that these factors were not considered in the decision-making process. Moreover, in one case, the truth was not told to the client. This was significantly different from what our IP-SDM model proposes: to actively seek values and preferences of the client and to foster a choice that is congruent with those. It seems that family caregivers wanted to protect their loved ones from making a difficult decision. The decision making process regarding location of care is clearly value laden [1] and thus requires a decision support intervention that will provide health professionals with the skills and tools they need to help clients and their family caregivers weigh options and clarify what is most important to them [37-39]. This will be crucial to stop the “silent misdiagnosis of clients’ preferences”, a key determinant of the performance of the healthcare system as a whole [40]. On the other hand, it is also possible that family caregivers felt that making a decision based on the therapeutic interests of the incapacitated person was a more realistic approach from a legal, ethical, and medical perspective [41]. “Therapeutic privilege” refers to the right of surrogate decision makers to not share with clients information that could harm them, or “to keep from clients information that could, for example, cause anxiety” [42]. In the context of decision making regarding location of care, it appears that family caregivers assumed this therapeutic privilege.

Fourthly, we observed that family caregivers felt some pressure to choose a specific option in a short period of time. This could be due to time constraints, which were reported as a barrier to implementation of IP-SDM by health professionals and managers of the IP teams. Family caregivers also felt some pressure to choose a specific option based on their financial means, i.e. to pay for private care. Although many factors influence the decision about location of care, some of which family caregivers, clients and IP home care team members may feel they cannot control [5], fostering IP-SDM may at least contribute to more realistic expectations about available options [42] and in turn lower decisional regret [43]. In addition, although much electronic information is available on the internet, it is not always reliable, and not all elderly people have access to the internet. Therefore, there is still a role to be played by health professionals in supporting the elderly in making difficult decisions.

Lastly, most family caregivers reported being supported by only one health professional (a social worker, as is usually the case in the home care system in the Province of Québec) and they did not feel they had experienced any IP-SDM. This could be partly due to the fact that they only saw one professional at a time and were not told that health professionals interact among themselves on their behalf but outside of their view. Although family caregivers reported a lack of interprofessional collaboration, that does not mean it was not taking place behind the scenes. In addition, they felt that the situation presented in the video should be a model used in home care. With the help of the video, they were in a better position to understand that a group of five diverse health professionals could work together as a team in the best interests of the clients using a common approach to the decision making process in an asynchronous manner. From an organizational point of view, it would be interesting to monitor interactions among health care professionals and communicate this to clients (i.e. a copy of the record could be left at the client’s home and the health professional could discuss it with the family). However, health professionals and managers reported that synchronous meetings of IP teams to discuss a case would facilitate the implementation of IP-SDM. Some might argue that if fewer health professionals were involved in SDM, or even just a case manager alone, the process would be both more efficient and more cost-effective. However, the key message is rather that for a specific decision making process, those who are involved should be attuned to an agreed upon decision making process.

Our study has limitations and strengths. First, it was an exploratory case study limited by constraints of the availability of the participants and the IP home care team. The fact that the clinical coordinator of the IP home care team selected the family caregivers for participating in this study could have introduced a source of bias. He could have suggested participants likely to have a more positive opinion of IP-SDM. The video that was presented could have influenced participants’ answers, although the differences between the video and participants’ real-life experience provided us with important information on the feasibility of IP-SDM. Also, we acknowledge that our own experience with aging parents may have influenced our interpretation of the data as most of team members involved in this study are involved with aging parents who are facing or will soon face the difficult decision to stay at home or move to a long term care facility. A strength of this study was the depth and rigour of our analysis of the transcripts: two team members independently analyzed them for themes, a third team member audited them, and the tape recordings ensured that none of the subtleties in the data were missed. Although the study was limited to one IP home care team in a city and therefore findings cannot be transferred to other geographical contexts, some of our results are validated by results of other research in this area. Participants in this study were selected not in order to constitute a representative sample of a target population, but in relation to the model of IP-SDM. Thus we are not in a position to pronounce on family caregivers’ situation overall.

## Conclusions

In this study, family caregivers described their experiences of the decision making process regarding location of care for their relatives receiving home care. They indicated that they did not experience IP-SDM when deciding to relocate their relative. Added to results obtained with health professionals and managers, our results highlight the need for an effective intervention targeting identified barriers to implementing IP-SDM in this context.

## Competing interests

The authors declare that they have no competing interests.

## Authors’ contributions

FL, DS, NB and SD conceptualized the study design. MCL, HR and FL conducted analyses. FL, HR and RD wrote the first draft of the paper. All authors critically revised the manuscript, read and approved the final version.

## Pre-publication history

The pre-publication history for this paper can be accessed here:

http://www.biomedcentral.com/1471-2318/14/83/prepub
